# Canine Leptospirosis in Flood-Affected Areas of Southern Brazil: Molecular Assessment and Public Health Implications

**DOI:** 10.3390/idr17030063

**Published:** 2025-06-03

**Authors:** Gabriela Merker Breyer, Nathasha Noronha Arechavaleta, Bruna Corrêa da Silva, Maria Eduarda Rocha Jacques da Silva, Mariana Costa Torres, Laura Cadó Nemitz, Rafaela da Rosa Marques, Fernando Borges Meurer, Gabriela Amanda Linden, Tainara Soares Weyh, Franciele Maboni Siqueira

**Affiliations:** 1 Laboratório de Bacteriologia Veterinária (LaBacVet), Departamento de Patologia Veterinária, Universidade Federal do Rio Grande do Sul, 9090/42704 Bento Gonçalves Avenue, Agronomia, Porto Alegre 91540-000, RS, Brazil; gabibreyer@hotmail.com (G.M.B.);; 2 Programa de Pós-Graduação em Ciências Veterinárias, Faculdade de Veterinária, Universidade Federal do Rio Grande do Sul, 9090/42704 Bento Gonçalves Avenue, Agronomia, Porto Alegre 91540-000, RS, Brazil

**Keywords:** *Leptospira*, One Health, climate change, *secY*, qPCR

## Abstract

Background: Southern Brazil faced massive rains and floods in May 2024, which led to social, infrastructural, and One Health issues affecting over 478 municipalities and 2.3 million people. Exposure to floodwater increased the risk of bacterial infections, including leptospirosis. Despite the zoonotic nature of leptospiral infections, only human leptospirosis is subject to mandatory reporting, while canine cases are less closely monitored. Considering the extent of this climatic event, many emergency shelters were created for rescued dogs, highlighting the need to monitor infectious diseases to mitigate the spread of hazardous pathogens. Methods: We performed a molecular assessment of canine leptospirosis in Porto Alegre and its metropolitan region. A total of 246 dogs rescued from the flooded areas underwent molecular diagnosis targeting *lipL32.* In addition, positive samples were identified by sequencing of the partial *secY* gene. Results: A total of 9 (4%) dogs were positive for *Leptospira* spp. Molecular and phylogenetic analyses of *secY* from the positive samples determined that the circulating strains belonged to *L. interrogans* (*n* = 8)—Icterohaemorrhagiae and Pomona as the suggested serogroups—and *L. kirschneri* (*n* = 1). Conclusions: Our findings point out the challenges in diagnosing and controlling leptospirosis during severe climatic events and reinforce the need for preventive sanitary measures to mitigate the dissemination of *Leptospira* spp., including the adoption of a mandatory notification system for canine leptospirosis.

## 1. Introduction

Leptospirosis is a zoonotic disease caused by *Leptospira* spp. with widespread occurrence in tropical and subtropical countries, particularly following periods of heavy rainfall and flooding [1]. Remarkably, climate change contributes to the emergence of bacterial infections, including leptospirosis, by increasing the frequency and severity of natural events that favor the survival of bacteria in the environment [2,3].

Leptospirosis pathogenesis includes an early and an acute stage of infection characterized by the circulation of the pathogen in the bloodstream (bacteremic phase) within the first 10 days of infection, followed by a late phase in which the pathogen translocates to the hosts’ tissues (icteric phase) [4]. Although *Leptospira* spp. cannot replicate outside a host, pathogenic serovars can remain viable in water and soil for weeks, serving as an indirect route of transmission to humans [5]. In addition, dogs can shed *Leptospira* spp. via urine even in the absence of clinical signs, posing a risk for direct zoonotic transmission of this disease [6].

From late April to May 2024, Rio Grande do Sul (Southern Brazil) faced heavy rains that caused massive flooding and damage across 478 municipalities, affecting over 2.3 million people [7]. This catastrophe caused a breakdown of the drainage and sanitation systems in the affected cities, increasing the risk of *Leptospira* spp. infections in both humans and animals. Emergency shelters were established to accommodate people and their pets after rescue; in addition, 85 dog shelters were registered in the capital, Porto Alegre, according to the Centro Estadual de Vigilância Sanitária (CEVS/RS), housing 5691 dogs for over two months. The lack of adequate infrastructure and overcrowding of the temporary dog shelters, together with the first reports of human leptospirosis cases related to the floods [8], raised a One Health concern regarding the spread of *Leptospira* spp.

Therefore, we performed a molecular assessment of canine leptospirosis in Porto Alegre and its metropolitan region, aiming to provide early diagnosis and mitigate further bacterial dissemination to other animals and humans during this climatic event. This descriptive study ultimately aims to reinforce the impacts of climate change on the spread of zoonotic diseases, and emphasizes the urgent need for mandatory notification of animal leptospirosis in Brazil.

## 2. Material and Methods

### 2.1. Animals and Sample Collection

Samples from 246 dogs rescued from the flooding areas in Porto Alegre and its metropolitan region were received at the laboratory for leptospirosis molecular diagnosis from 16 May to 24 June 2024. All animals were handled by registered veterinarians following the standards for the care and use of animals; therefore, no ethical approval was required for the molecular detection of *Leptospira* spp. performed in this study.

**Figure 1:**
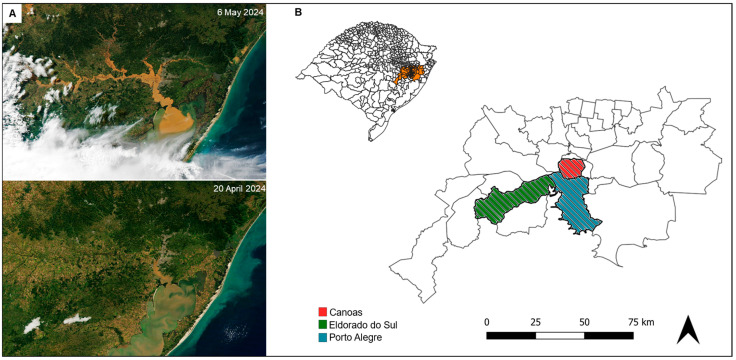
Flood impact in the Metropolitan Region of Porto Alegre. (**A**) Satellite view of the region before (20 April 2024), and after (6 May 2024) the floods, adapted from NASA’s International Space Station Program [9]. (**B**) Distribution of the analyzed dogs according to their rescue location in Porto Alegre and its metropolitan region (orange). The map in the upper left corner shows the state of Rio Grande do Sul, Brazil. Maps were generated using QGIS 3.38.0 [10].

All dogs included in the study were rescued from flood-affected areas, were potentially exposed to contaminated water, and were housed in emergency shelters or temporary homes during sampling in Porto Alegre and its metropolitan region (Figure 1). From all 246 dogs examined in this study, information on gender, age, rescue location, and clinical evaluation was gathered. The clinical history and rescue location of these animals were provided by the veterinarians, and the dogs were classified as suspected and asymptomatic for leptospirosis based on this information. In detail, dogs exhibiting any of the following clinical signs were classified as suspected for leptospirosis: loss of appetite, vomiting, lethargy, abdominal pain, diarrhea, jaundice, dehydration, polyuria or polydipsia, weight loss, stiffness, or muscle pain. As there are no pathognomonic signs for leptospirosis, the animals’ clinical history was recorded solely to aid the interpretation of our findings.

Given the pathogenesis of leptospirosis and the unknown background of the animals, both urine and blood samples were collected when available to ensure accurate diagnosis. Urine samples were obtained through spontaneous urination into sterile collection containers, while blood samples were collected from the cephalic or jugular veins using EDTA blood collection tubes. All samples obtained from the rescued dogs were stored at 4 °C and analyzed within 48 h of collection.

### 2.2. In Vitro Specificity of Leptospira spp. Detection in Blood and Urine Samples

To ensure efficient detection in clinical specimens, non-infected samples were experimentally contaminated with different clinical *Leptospira interrogans* serovars (Canicola, Copenhageni, and Pomona). Pure leptospiral cultures were performed and kindly provided by the Laboratório de Vacinologia—Universidade Federal de Pelotas (UFPEL).

In vitro contamination was performed by inoculating each *Leptospira* sp. serovar (10^7^ cell density/mL) into non-infected samples (blood, urine, and pooled blood and urine) obtained from a healthy animal. For blood contamination, the bacterial suspension was added to 200 µL of non-infected blood. For urine contamination, 1 mL of non-infected urine was harvested by centrifugation (8000× *g* for 10 min) and resuspended in 200 µL PBS 1X, then inoculated with the bacterial suspension. Finally, for pooled samples, 1 mL of non-infected urine was harvested by centrifugation (8000× *g* for 10 min) and resuspended in 200 µL of non-infected blood. In addition, a 1 mL aliquot of each culture was harvested (12,000× *g* for 10 min), resuspended in 200 µL PBS 1X, and subjected to DNA isolation.

### 2.3. qPCR Validation and Detection of Leptospira spp. in Canine Clinical Samples

Pure cultures and in vitro contaminated samples were subjected to DNA isolation by the DNA Mini Kit Blood/Tissue (Mebep Bioscience, Shenzhen, China), according to the manufacturer’s instructions.

Canine urine and blood samples were pooled for further analysis. Briefly, 1 mL of urine was harvested at 8000× *g* for 10 min; the pellet was resuspended in 100 µL PBS 1X, then added to 100 µL of blood and gently homogenized. This procedure was performed for each sample/animal analyzed in this study, followed by DNA extraction using the DNA Mini Kit Blood/Tissue (Mebep Bioscience, China).

*Leptospira* spp. detection was performed using the commercial product LPTAmp (Simbios Biotecnologia, Cachoeirinha, Brazil), which targets the *lipL32* gene, following the manufacturer’s instructions on the QuantStudio™ 3 Real-Time PCR System (Applied Biosystems, Waltham, MA, USA). Reactions were performed in duplicate, and each assay included positive and negative controls using the commercial product DNA LPTAmp Ref (Simbios Biotecnologia, Brazil) and ultrapure water, respectively.

Firstly, the assay’s ability to detect different serovars was assessed using DNA templates from *L. interrogans* Canicola, Copenhageni, and Pomona, including both pure culture and in vitro contaminated specimens. In addition, to determine the assay’s sensitivity, a standard curve was generated using 10-fold serial dilutions of DNA isolated from urine and blood pools contaminated with *L. interrogans* Canicola. For bacterial quantification, a linear regression was calculated based on Ct values and copy number, which was estimated using the DNA Copy Number and Dilution Calculator tool (Thermo Fisher Scientific, Waltham, MA, USA), considering an average *Leptospira* spp. genome size of 4.3 Mb. The amplification factor and efficiency were calculated using the qPCR Efficiency Calculator (Thermo Fisher Scientific, USA).

For the analysis of canine clinical samples, the baseline and threshold were set manually (baseline from cycles 1 to 19; ΔRn = 10,000).

### 2.4. Leptospira spp. Differentiation Based on Partial secY Sequencing

*Leptospira* spp.-positive samples were subjected to *secY* amplification for further bacterial differentiation and phylogenetic analysis. In addition, the pure leptospiral culture samples used in the qPCR validation were included in this analysis. Nested PCR targeting a partial *secY* gene fragment was performed as follows: (i) The first reaction amplified a 549 bp gene fragment in 25 µL volume containing 1 U Taq DNA polymerase recombinant (Thermo Fisher Scientific, USA), 1 mM dNTPs, 1× buffer, 1 mM MgCl_2_, 0.2 µM of each primer (5′-ATGCCGATCATTTTTGCTTC-3′, and 5′-CCGTCCCTTAATTTTAGACTTCTTC-3′) [11], and 50 ng of DNA. Cycle conditions included an initial denaturation at 95 °C for 5 min, followed by 35 cycles of 94 °C for 30 s, 52 °C for 30 s, and 72 °C for 1 min. (ii) The second reaction amplified a 410 bp fragment in 50 µL containing 1 U Taq DNA polymerase recombinant (Thermo Fisher Scientific, USA), 1 mM dNTPs, 1× buffer, 1 mM MgCl_2_, 0.2 µM of each primer (5′-CCTCAGACGATTATTCAATGGTTATC-3′, and 5′-AGAAGAGAAGTTCCACCGAATG-3′) [12], and 2 µL amplicon from the first reaction. Cycle conditions were the same as in the first reaction, except for an increase in the melting temperature (Tm) to 54 °C. PCR products were evaluated on a 1% agarose gel stained with 32 × Unisafe dye (UniScience Corporation, Hialeah, USA). Final amplification products were purified using the PureLink Quick PCR Purification kit (Thermo Fisher Scientific, USA) and subjected to Sanger sequencing. Partial *secY* sequences were de novo assembled using Geneious Prime 2024.2.24 [13] and compared against the National Center for Biotechnology Information (NCBI) database using BLASTn.

To determine the leptospiral species and infer serogroups, partial *secY* sequences from strains in this study were compared with 41 additional *secY* sequences of leptospiral strains with known serovars retrieved from the GenBank database—NCBI (Supplementary Table S1). Sequence alignment was performed using ClustalW, and the phylogenetic tree was built by neighbor-joining (bootstrap = 1000) using the Tamura-3-parameter model (gamma = 0.2506).

## 3. Results

### 3.1. Validation of Pooled Samples for Molecular Diagnosis of Leptospirosis

The *Leptospira* spp. qPCR assay successfully detected all clinical serovars tested in this study—*L. interrogans* Canicola, Copenhageni, and Pomona—in both pure cultures and in vitro contaminated clinical specimens (urine, blood, and pool).

Bacterial quantification was estimated using the standard curve generated from in vitro contaminated pooled samples, with a detection range from 2 to 3.8 × 10^6^ copies/µL. No amplification was observed beyond the 10^−6^ dilution in the qPCR assay. Linear regression based on the serial dilutions was calculated as follows: y = −0.928x + 25.512, where *y* represents log_10_ (copy number) and *x* corresponds to the observed Ct (R^2^ = 0.957; efficiency = 747.23%).

### 3.2. Occurrence of Leptospira spp. in Dogs Rescued from the Floods in Porto Alegre and Metropolitan Region

Available data on the 246 dogs included in this study are presented in Supplementary Table S2. Most rescued dogs were mixed breed (91%), male (63%), and adults (72%). Among the tested animals, 35 dogs (14%) exhibited clinical signs indicative of leptospirosis, including lethargy (14/35), loss of appetite (11/35), diarrhea (11/35), jaundice (9/35), dehydration (5/35), vomiting (5/35), weight loss (2/35), abdominal pain (2/35), polyuria or polydipsia (1/35), stiffness or muscle pain (1/35), and hematuria (1/35); these animals were classified as suspected cases. The remaining 211 animals (86%) showed none of the listed clinical signs and were classified as asymptomatic. In addition, 19 dogs (7%) received antibiotic treatment; 11 of which presented the aforementioned clinical signs, and eight were asymptomatic and treated for unrelated reasons.

Molecular detection of *Leptospira* spp. was observed in nine dogs (4%) with variable bacterial loads ranging from 2 to 2.23 × 10^9^ copy numbers (Table 1). Notably, no *Leptospira* spp. was detected in 23 (10%) of the tested animals classified as clinically suspected.

The positive animals were rescued from Porto Alegre (5/9), Canoas (1/9), and Eldorado do Sul (1/9), whereas two dogs were originated from undetermined locations. Based on clinical history, only two dogs (030/24-5 and 050/24) exhibited signs suggestive of leptospirosis and had already received doxycycline treatment at the time of sampling. In addition, two other asymptomatic dogs (047/24-1 and 060/24-7) had been treated with antibiotic for unrelated reasons (Table 1).

### 3.3. Leptospira spp. Differentiation Based on secY Phylogenetic Analysis

**Figure 2:**
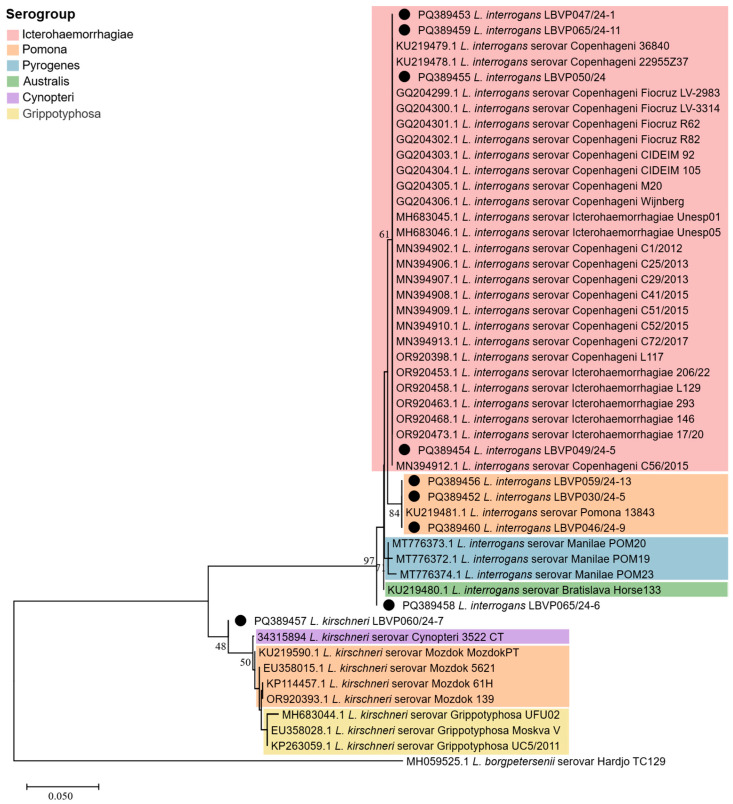
Phylogenetic analysis of partial *secY* sequences from *Leptospira* spp. detected in canine samples during the 2024 floods in Rio Grande do Sul, Brazil. The tree was built using the neighbor-joining method (boostrap = 1000) with the Tamura Nei model (gamma = 0.2509). Sequences obtained in this study are indicated (●). Serogroups are grouped by colors: Icterohaemorrhagiae (red), Pomona (orange), Pyrogenes (blue), Australis (green), Cynopteri (purple), Grippotyphosa (yellow). Only the strains from this study exhibited 100% *secY* identity with previously deposited leptospiral strains were classified at the serogroup level.

Based on partial *secY* sequencing followed by BLASTn search, we identified eight *L. interrogans* strains (LBVP030/24-5, LBVP046/24-9, LBVP047/24-1, LBVP049/24-5, LBVP050/24, LBVP059/24-13, LBVP065/24-6, and LBVP065/24-11) and one *L. kirschneri* strain (LBV060/24-7), with high identities (98.54-100%). In addition, we built a phylogenetic tree to assess the intraspecific diversity of strains circulating in Porto Alegre and its metropolitan region in an attempt to infer leptospiral serogroups based on evolutionary distances from reference isolates (characterized by MAT and retrieved from the NCBI database; Figure 2). Only strains with 100% *secY* identity to previously deposited leptospiral strains were assigned to a specific serogroup. Within the *L. interrogans* clade, a subclade corresponding to the Icterohaemorrhagiae serogroup was identified, comprising strains LBVP047/24-1, LBVP049/24-5, LBVP050/24, and LBVP065/24-11. In addition, *L. interrogans* strains LBVP030/24-5, LBVP046/24-9, and LBVP059/24-13 showed high similarity to *L. interrogans* 13843, suggesting affiliation with Pomona serogroup. No clear clustering was observed for *L. interrogans* LBVP065/24-6, which may indicate affiliation with a serogroup not represented in our dataset. Meanwhile, the *L. kirschneri* strain LBV060/24-7 clustered with other *L. kirschneri* strains but was distant from the Cynopteri, Pomona, and Grippotyphosa serogroups, suggesting it belongs to a different serogroup.

## 4. Discussion

The catastrophic floods in Southern Brazil highlight the urgent need to implement preventive and mitigation strategies for climate-related events, encompassing infrastructure, social, and sanitary dimensions [14]. In this study, we raised a One Health concern regarding canine leptospirosis cases in emergency shelters in Porto Alegre and its metropolitan region. We validated a qPCR assay for leptospiral detection using pooled samples (blood and urine), which confirmed nine cases of canine leptospirosis, most of which involved asymptomatic dogs. Despite the zoonotic risk posed by untreated dogs, animal leptospirosis is not a notifiable disease in Brazil. This contributes to data underreporting and, consequently, hinders the implementation of effective measures for disease control.

Moreover, although the diagnosis of leptospirosis remains challenging, several diagnostic methods have been described in the literature, including direct visualization by darkfield microscopy, bacterial culture, tissue biopsy, molecular-based approaches, and serological methods, such as the enzyme-linked immunosorbent assay (ELISA), and the microscopic agglutination test (MAT) [6]. Direct visualization and culture require long incubation periods and may have low specificity; ELISA is faster but shows low sensitivity and specificity. Therefore, current confirmatory methods for early detection of *Leptospira* spp. rely on MAT and PCR, preferably in combination [15]. MAT is the reference method for the serological diagnosis of leptospirosis in dogs; however, it is a laborious method and may not be accurate during the first days of infection [6]. Given the catastrophic scenario in Porto Alegre and its metropolitan area, only a limited number of laboratories were available for MAT detection due to disrupted transport routes during the floods. Also, MAT results would likely have low accuracy because there was no clear timeline of the animals’ exposure to contaminated floodwaters. Additionally, dogs are considered one of the main sources of leptospirosis transmission to humans [14], as they can continuously shed the bacteria through urine, even in the absence of clinical signs [16]. Thus, considering the One Health emergency during the floods in Rio Grande do Sul, PCR was the most feasible method for early detection of *Leptospira* spp. in rescued dogs, allowing early treatment and preventing the spread of the pathogen to other dogs and humans.

*Leptospira* spp. molecular detection can be performed on blood specimens during the initial stage of infection—within the first 10 days (bacteremia)—and on urine samples after the first week of infection (bacteriuria) [6]. Considering that the timeline of infection in the analyzed animals was usually unknown, we preferentially analyzed both blood and urine samples from each animal, when available. We validated the qPCR assay using pooled samples by analyzing experimentally infected pools with *L. interrogans* Canicola, which showed a high sensitivity range. Although combining blood and urine samples could dilute the pathogen concentration in the assay, this approach offers an accurate diagnostic strategy with reduced costs and increased likelihood of detection in animals lacking a clear history of exposure to contaminated floodwaters.

According to qPCR analysis, *Leptospira* spp. were detected in 4% of the rescued dogs in this catastrophic scenario. A prospective study investigating the occurrence of leptospirosis in sheltered dogs in Brazil reported that 10.6% tested positive for *Leptospira* spp., based on both PCR and MAT methods [15]. A review of the epidemiological situation of leptospirosis in stray and sheltered dogs pointed out a high variability in the proportion of positive animals based on MAT and PCR diagnostics, with Canicola, Icterohaemorrhagiae, Grippotyphosa, and Pomona being the most commonly identified serovars [17]. Another study conducted in the municipality of Porto Alegre between 2007 and 2009 investigated leptospiral infection in 253 dogs through PCR and MAT, which determined the presence of *Leptospira* spp. in 14% and 48%, respectively, demonstrating substantial differences in detection rates between molecular and serological methods [17]. Similar discrepancies were observed in a study conducted in Malaysia on the early diagnosis of leptospirosis in humans, in which positivity was detected in 26% of patients by MAT, and in 38% by PCR, with only 8% of samples testing positive by both methods; thus, combined molecular and serological methods are suggested for early leptospirosis diagnosis, regardless of the infection period [15]. We highlight that our results should be interpreted with caution, as leptospirosis transmission during a catastrophic event may differ from that under natural conditions; however, this disease is well known to be associated with flood events. Nevertheless, considering previous data indicating a 14% occurrence of canine leptospirosis in Porto Alegre based on PCR assay [18] and the total number of rescued dogs during this event (*n* = 5691), the sample group included in this study (*n* = 246) is representative of the affected population. Moreover, during this climate event, many shelters adopted antibiotic prophylaxis as part of their routine to prevent bacterial infections among rescued dogs, which may have interfered with molecular diagnosis of leptospirosis. The use of antibiotics could explain the absence of *Leptospira* detection in 23 suspected cases included in the present study. Regarding the bacterial load found in the positive animals (*n* = 9), it ranged from 2 to 2.23 × 10^9^ copies/µL, which may reflect the stage of infection at the time of sampling (bacteremic or icteric phase), as well as the type of sample collected. Notably, leptospiral shedding in canine urine may be intermittent. Noteworthy, most of the positive animals were asymptomatic, suggesting no clear correlation between bacterial load and clinical signs.

As canine leptospirosis is usually caused by *L. interrogans*, *L. borgpetersenii*, and *L. kirschneri* [19], we performed bacterial identification based on the *secY* gene and identified two leptospiral species circulating in the flooded area: *L. kirschneri* (n = 1) and *L. interrogans* (*n* = 8). Unfortunately, due to the unavailability of serological testing, a molecular approach based on *secY* phylogenetic analysis was used to assess intraspecific diversity among the *Leptospira* spp. in Porto Alegre and its metropolitan region, aiming to infer potential serogroups through comparison with existing data. This analysis suggested that Icterohaemorrhagiae (*n* = 4) and Pomona (*n* = 3) serogroups, both showing 100% identity with *secY* sequences from classical leptospiral isolates, were the most likely to be circulating during the floods. Notably, these findings are genetic inferences based on *secY*, a gene proposed as a genetic marker for leptospiral intraspecific diversity [20,21], but further serological analyses are required to confirm the specific serovars and serogroups involved.

## 5. Conclusions

Despite the relatively low prevalence of canine leptospirosis among the dogs rescued during the floods in Rio Grande do Sul, the molecular detection of *Leptospira* spp. allowed early diagnosis of the disease, which was essential to prevent its spread to other animals and humans. Furthermore, we validated the qPCR method using pooled blood and urine samples, allowing for effective diagnosis regardless of the stage of infection through a single analysis. Overall, we emphasize the One Health concern posed by leptospirosis in the context of climate disasters and highlight the need for preventive sanitary measures to mitigate the spread of *Leptospira* spp. in both animals and humans, perhaps adopting a mandatory notification system for such diseases in animals.

## Figures and Tables

**Table 1:** Animals with leptospirosis based on qPCR assay.

Animal ID	Breed	Lifecycle Stage	Gender	Rescue Location (City)	Clinical Signs		Treatment		Molecular Analysis		
					Status	Description	Any Antibiotic Therapy? *	Antimicrobial Drug (days)	Sample Type	CT	Leptospiral Identification **
030/24-5	Mixed breed	Senior	Female	Eldorado do Sul	Suspected	Lethargy/Jaundice	Yes	doxycycline (3)	Blood	35.69	*L. interrogans*
046/24-9	Mixed breed	unknown	Male	Canoas	Asymptomatic		No		Blood	34.67	*L. interrogans*
047/24-1	American Pit Bull Terrier	Puppy	Male	Porto Alegre	Asymptomatic		Yes	doxycycline (12)	Urine	25.89	*L. interrogans*
049/24-5	Mixed breed	unknown	Female	Porto Alegre	Asymptomatic		No		Pool	33.31	*L. interrogans*
050/24	Mixed breed	Adult	Male	Porto Alegre	Suspected	Jaundice	Yes	doxycycline (3)	Urine	32.48	*L. interrogans*
059/24-13	Mixed breed	Adult	Male	Porto Alegre	Asymptomatic		unknown		Pool	18.33	*L. interrogans*
060/24-7	Mixed breed	Adult	Male	unknown	Asymptomatic		Yes	enrofloxacin (3)	Urine	27.08	*L. kirschneri*
065/24-6	Mixed breed	Adult	Male	unknown	Asymptomatic		No		Urine	35.80	*L. interrogans*
065/24-11	Mixed breed	Adult	Male	Porto Alegre	Asymptomatic		No		Urine	38.66	*L. interrogans*

* prior to the sampling; ** based on *secY* sequecing.

## Data Availability

Data available on request.

## Supplementary Materials

The following supporting information can be downloaded at: https://www.mdpi.com/article/10.3390/idr17030063/s1, Table S1: Description of leptospiral strains used for secY phylogenetic analysis; Table S2: General data of the rescued dogs analyzed for leptospirosis.
